# Multiple brain abscesses with good prognosis in an infant with cyanotic congenital heart disease: a case report

**DOI:** 10.1186/s13256-020-02436-3

**Published:** 2020-07-21

**Authors:** Atsuko Kudo-Kubo, Shuichi Shimakawa, Yutaka Odanaka, Naokado Ikeda, Hikaru Kitahara, Hiromitsu Toshikawa, Atsuko Ashida, Miho Fukui, Noriyasu Ozaki, Kanta Kishi, Masahiko Wanibuchi, Akira Ashida

**Affiliations:** 1 Department of Pediatrics, Osaka Medical College, 2-7 Daigaku-machi, Takatsuki, Takatsuki, Osaka, 569-8686 Japan; 2 Department of Neurosurgery, Osaka Medical College, Takatsuki, Osaka, Japan

**Keywords:** Brain abscess, Multiple, Cyanotic congenital heart disease, Children

## Abstract

**Background:**

Brain abscesses are relatively rare, but they are a potentially life-threatening condition. Predictive factors for poor outcome are a young age and the presence of multiple abscesses. We report a case of a 15-month-old girl with cyanotic congenital heart disease who developed multiple brain abscesses caused by *Streptococcus intermedius.* The patient was treated with a combination of surgical aspiration and antimicrobial therapy without apparent neurological sequelae. To the best of our knowledge, this is the youngest such patient to have been reported in the literature. We explore the possible causes of her good outcome.

**Case presentation:**

At the age of 15 months, the Japanese patient initially was presented to our hospital with transient eye deviation to the left and vomiting. In a blood examination, her white blood cell count (12,720 per mm^3^ with a left shift) and C-reactive protein level (1.23 mg/ml) were slightly elevated. Magnetic resonance imaging of the brain showed three mass lesions. These were 1.5-cm, 1.9-cm, and 1.2-cm rim-enhancing lesions with extensive surrounding edema. Brain abscesses were diagnosed, and vancomycin (50 mg every 12 hours) and meropenem (40 mg every 8 hours) were started empirically. However, because each brain abscess was enlarged at 8 days after admission, surgical aspiration was performed at 10 days after admission, and cultures of the aspirated pus grew *S. intermedius*. Penicillin G (0.7 million units every 4 hours) and ceftriaxone (280 mg every 12 hours), to which this isolate is susceptible, were then administered, and the brain abscesses reduced in size. After 1 month of ceftriaxone and 3 months of penicillin G treatment, all of the brain abscesses disappeared. Apparent neurological sequelae were not observed at 6 months after onset.

**Conclusions:**

A good outcome can be obtained if multiple brain abscesses develop in infancy or early childhood in cases without unconsciousness at admission, meningitis, or sepsis. Appropriate antimicrobial therapy should be started immediately after diagnosis, with surgical aspiration performed to identify the causative pathogen and avoid intraventricular rupture of the brain abscesses.

## Background

Brain abscesses occur infrequently, with an incidence of 4 cases per 1 million [1]; however, they are a potentially life-threatening condition [2]. In children, mortality has been reported as 3.7–24%, and 19–34.8% of patients have neurological sequelae [3, 4]. According to past reports, predictive factors for poor outcome are age younger than 5 years and the presence of multiple abscesses [1, 3].

Cyanotic congenital heart disease (CCHD) is the most common predisposing factor for brain abscess development. The number of patients with brain abscess who are older than 10 years of age is currently decreasing [1, 3]; thus, the age of onset of brain abscesses might be becoming younger. Brain abscesses in patients with CCHD tend to present as multiple lesions because a hematogenous spread of bacteria is associated with CCHD [5]. On the basis of the above, brain abscess in patients with CCHD might have a worse prognosis. Therefore, the prognosis of brain abscess in patients with CCHD needs to be improved. However, there are currently no effective prophylactic therapies or guidelines for the management of brain abscess in children. Reports of patients with good outcomes may therefore be important for improving patient prognosis.

We report a case of a 15-month-old girl with CCHD who developed multiple brain abscesses caused by *Streptococcus intermedius* that were treated with a combination of surgical aspiration and antimicrobial therapy. To the best of our knowledge, she is the youngest reported patient with CCHD to develop multiple brain abscesses caused by *S. intermedius*, and she was successfully treated without apparent neurological sequelae. We consider the possible causes of this good outcome.

## Case presentation

**Fig. 1:**
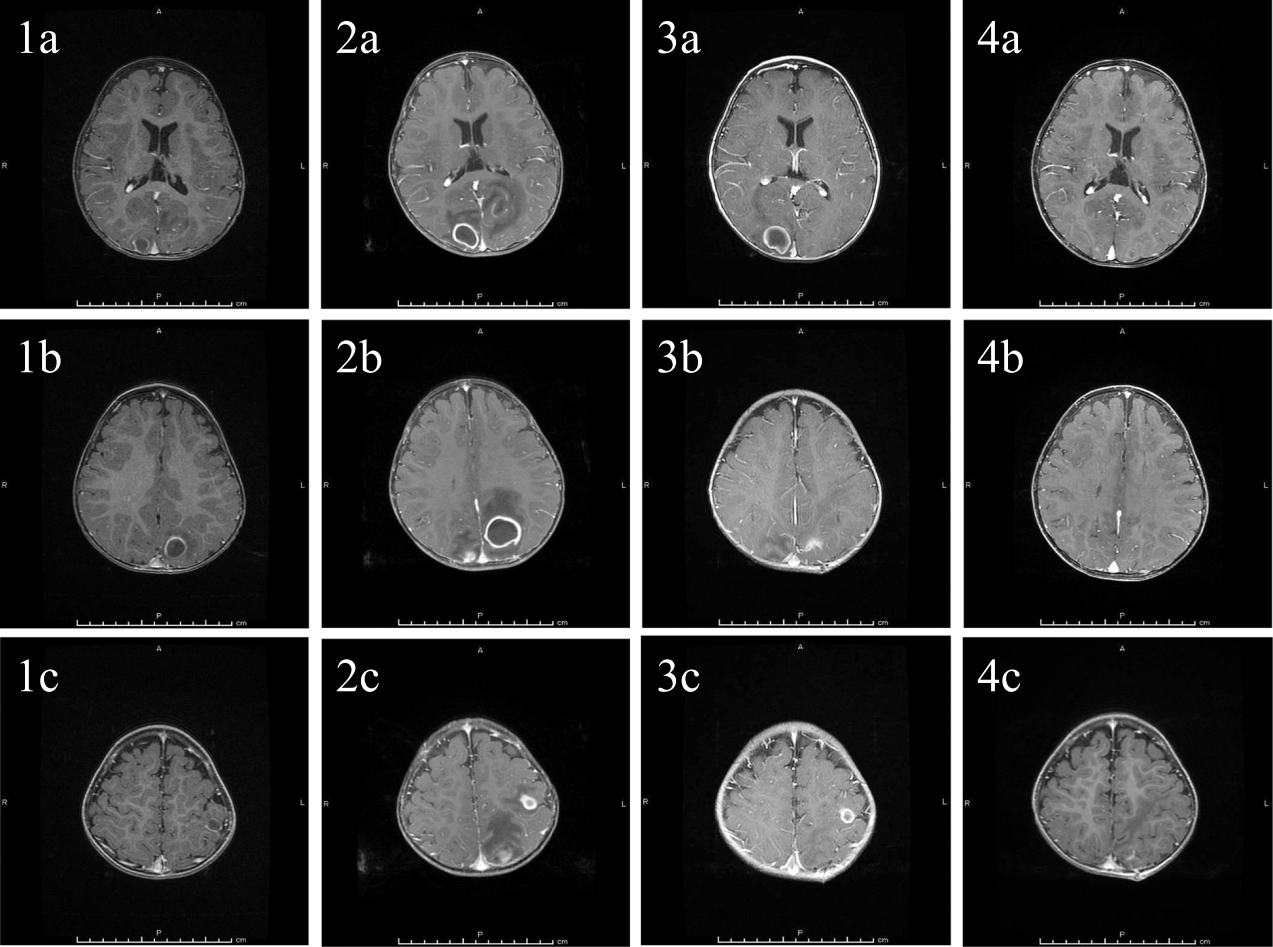
Clinical course of the lesions as seen on gadolinium-enhanced T1-weighted magnetic resonance images. Brain abscesses were located in the right occipital lobe (1a–4a), left occipital lobe (1b–4b), and left parietal lobe (1c–4c) on the day of admission (1a–1c), at 8 days after admission (2a–2c), at 13 days after surgical aspiration (3a–3c), and at 4.5 months after onset (4a–4c). Each brain abscess was enlarged after admission (2a–2c), reduced after surgical aspiration (3a–3c), and eventually disappeared (4a–4c)

**Fig. 2:**
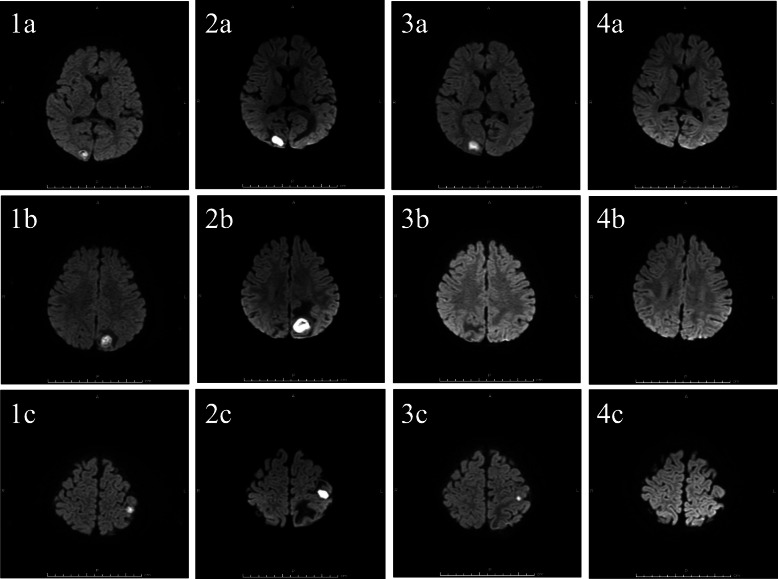
Clinical course of the lesions as seen on diffusion-weighted magnetic resonance images on the day of admission (1a–1c), at 8 days after admission (2a–2c), at 13 days after surgical aspiration (3a–3c), and at 4.5 months after onset (4a–4c). Very hyperintense lesions were revealed in the right occipital lobe (1a–4a), left occipital lobe (1b–4b), and left parietal lobe (1c–4c) on the day of admission (1a–1c). These lesions all enlarged before surgical aspiration (2a–2c). The left occipital lesion disappeared immediately (3b), and the other lesions reduced after surgical aspiration (3a, c) and eventually disappeared (4a, c)

Our patient was a 15-month-old Japanese girl with CCHD (complete transposition of the great arteries, bilateral pulmonary artery stenosis, and ventricular septal defect), cleft lip and palate, and duplicate ureter. In our hospital, she had a balloon atrioseptostomy at the age of 5 days, bidirectional Glenn surgery and a Damus–Kaye–Stansel anastomosis at the age of 4 months for her CCHD, and cleft lip plastic surgery for her cleft lip and palate at the age of 3 months. At age of 15 months, transient eye deviation to the left and vomiting were present once per day for 2 consecutive days, and she visited our hospital on day 2. She had no history of falls or trauma, and she did not have dental caries or otitis media. She was standing without support and could speak. An examination showed normal body temperature (37.6 °C) and blood pressure (88/46 mmHg). The patient’s heart rate (138 beats/minute) and respiration rate (38 breaths/minute) were slightly elevated. Her oxygen saturation (83% on room air) was reduced, as usual. A neurological examination showed generalized hyperreflexia. On admission to our hospital, a computed tomographic scan of the patient’s head showed a hypodense mass (1.5 cm in diameter) with adjacent edema in the left occipital lobe. In a blood examination, her white blood cell count (12,720 per mm^3^ with a left shift) and C-reactive protein level (1.23 mg/ml) were slightly elevated. A lumbar puncture was performed, and cerebrospinal fluid analysis revealed a colorless, transparent fluid with a white cell count of 2 per mm^3^ and 22.5 mg/dl of protein. Magnetic resonance imaging of the brain showed three mass lesions. These were rim-enhancing lesions with hyperintense signal on a diffusion-weighted image and extensive surrounding edema, and they had diameters of 1.5 cm, 1.9 cm, and 1.2 cm in the right occipital, left occipital, and left parietal lobe areas, respectively. Imaging features suggested that these lesions were abscesses (Figs. 1 and 2). Vancomycin (50 mg every 12 hours) and meropenem (40 mg every 8 hours) were started empirically.

Within the first 48 hours, the patient’s temperature was elevated to 39.5 °C and then subsided. Blood and cerebrospinal fluid cultures, taken on admission, did not grow any fungi. Because each brain abscess was enlarged at 8 days after admission, surgical aspiration was performed on day 10 after admission, and cultures of the aspirated pus were found to grow *S. intermedius* using the API Rapid ID 32 STREP System (bioMérieux, Marcy l’Etoile, France). Penicillin G (0.7 million units every 4 hours) and ceftriaxone (280 mg every 12 hours), to which this isolate is susceptible, were then administered, and the brain abscesses reduced in size. After 1 month of ceftriaxone and 3 months of penicillin G, all of the brain abscesses disappeared. The results of immunological function analysis were normal (serum immunoglobulin G, 319 mg/dl; CD4/CD8 ratio, 2.88; neutrophil phagocytosis function, 97.1%; and sterilization ability, 84.5%).

At 2.5 months after admission, the patient’s developmental quotient was measured using the Kyoto Scale of Psychological Development. Her developmental level for the cognitive–adaptive domain (nonverbal reasoning or visuospatial perception assessed using materials [for example, blocks, miniature cars, marbles]) was 80, and for the language–social domain (interpersonal relationships, socialization, and verbal abilities), it was 99. The patient’s Postural–Motor domain (fine and gross motor function) level was not measured, because she was hospitalized.

## Discussion and conclusions

We report a case of a 15-month-old girl with CCHD who developed multiple brain abscesses caused by *S. intermedius* that were successfully treated without apparent neurological sequelae. To the best of our knowledge, she is the youngest reported patient with CCHD to develop multiple brain abscesses caused by *S. intermedius*.

In patients with CCHD, the incidence of brain abscess is declining. According to an investigation in Boston, which compared patients treated between the years 1981–2000 and 1945–1980, CCHD is the most common predisposing factor for brain abscess; however, the percentage of the total has decreased from 50% to 25% [6]. In a 2010 investigation in Israel, the most common predisposing factors for brain abscess were reported to be sinusitis, meningitis, and traumatic brain injury [4]. The number of patients older than 10 years of age with brain abscess is decreasing, and early surgical interventions in developed countries may be causing this decline in incidence. However, in patients in early childhood and infancy, the incidence might not have changed. Felsenstein *et al.* reported that children with brain abscesses who had a neurological deficit after 6 months of age were younger than those who fully recovered and that an age younger than 5 years was associated with a poor outcome [3]. Brain abscesses in patients with CCHD tend to present as multiple lesions, because a hematogenous spread of bacteria is associated with CCHD [5]. It has been reported that many of the complications and deaths in children with brain abscesses can be attributed to multiple abscesses [3].

The reasons for our patient’s good outcome were explored. In previous papers, potential predictors of poor outcome in children with brain abscesses, other than a younger age and multiple lesions, have been reported as follows: (1) a lower level of consciousness at admission [3], (2) the development of meningitis [7], (3) antimicrobial therapy not started immediately after diagnosis, (4) lesions localized near ventricles or large in size, and (5) surgical aspiration is not implemented.

Felsenstein *et al.* reported that a Glasgow Coma Scale score < 8 at admission was associated with poor outcome in children with brain abscesses [3]. Furthermore, Ciurea *et al*. reported that the rate of disability in children with brain abscesses resulting from CCHD was lower than that in children with brain abscesses resulting from sepsis or meningitis [7]. Our patient’s level of consciousness was normal at admission, and she did not develop sepsis or meningitis.

A delay in the initiation of antimicrobial therapy can result in a poor outcome, as indicated by a retrospective study in which the median interval between diagnosis and the start of antimicrobial therapy was 2 days. In our patient, both diagnosis and the initiation of antimicrobial therapy occurred on the day she visited our hospital. Cephalosporin and metronidazole or meropenem are recommended as empirical treatments for brain abscesses, and vancomycin should also be added if a staphylococcal infection is suspected [5]. Because *Staphylococcus* and *Streptococcus* spp. are the most frequently isolated microbes in previous reports of patients with CCHD, appropriate antimicrobial therapy was started early in our patient.

Regarding the location and size of brain abscesses, in a previous Japanese report, intraventricular rupture of brain abscess (IVROBA) was a strong risk factor for poor outcome in 62 patients with CCHD in a multivariate logistic analysis [8]. Brain abscess that measures at least 1 cm in diameter is amenable to surgical aspiration [5]. In our patient, the lesion was located far from the lateral ventricles and measured more than 1 cm in diameter. Surgical aspiration could be performed, and enlargement of the abscesses was thus prevented. The authors of the previous Japanese report recommended that IVROBA should be aggressively treated with a combination of aspiration and microbial therapy.

Surgical aspiration is vital to identify the causative pathogen and avoid IVROBA [5, 8]. Hematoma and rupture of the abscess into the ventricles can occur as surgical complications [4, 6]. In our patient, the brain abscesses decreased in size after surgical aspiration, and *S. intermedius* was able to be isolated from pus obtained by surgical aspiration. In addition, no surgical or postsurgical complications occurred.

Our patient had no apparent neurological sequelae, although we have as yet only conducted follow-up of her for 6 months, and higher brain dysfunction and learning difficulty were not able to be evaluated. In a previous study, Carey *et al.* reported that, when tested 6 years after treatment, 70% of children had impaired scholastic ability [9]. The development of our patient thus needs to be followed up long-term.

In conclusion, our patient was a 15-month-old girl with CCHD who developed multiple brain abscesses, which were treated with a combination of surgical aspiration and antimicrobial therapy, without apparent neurological sequelae. We conclude that, even if multiple brain abscesses develop in infancy or early childhood, a good outcome may be obtained in cases without unconsciousness at admission, meningitis, or sepsis. Appropriate antimicrobial therapy should be started immediately after diagnosis, with surgical aspiration performed to identify the causative pathogen while avoiding IVROBA.

## Data Availability

Not applicable.

## Abbreviations

CCHD: Cyanotic congenital heart disease
IVROBA: Intraventricular rupture of brain abscess
